# Are There Side Effects to Watching 3D Movies? A Prospective Crossover Observational Study on Visually Induced Motion Sickness

**DOI:** 10.1371/journal.pone.0056160

**Published:** 2013-02-13

**Authors:** Angelo G. Solimini

**Affiliations:** Department of Public Health and Infectious Diseases, Sapienza University of Rome, Rome, Italy; California Pacific Medical Center Research Institute, United States of America

## Abstract

**Background:**

The increasing popularity of commercial movies showing three dimensional (3D) images has raised concern about possible adverse side effects on viewers.

**Methods and Findings:**

A prospective carryover observational study was designed to assess the effect of exposure (3D vs. 2D movie views) on self reported symptoms of visually induced motion sickness. The standardized Simulator Sickness Questionnaire (SSQ) was self administered on a convenience sample of 497 healthy adult volunteers before and after the vision of 2D and 3D movies. Viewers reporting some sickness (SSQ total score>15) were 54.8% of the total sample after the 3D movie compared to 14.1% of total sample after the 2D movie. Symptom intensity was 8.8 times higher than baseline after exposure to 3D movie (compared to the increase of 2 times the baseline after the 2D movie). Multivariate modeling of visually induced motion sickness as response variables pointed out the significant effects of exposure to 3D movie, history of car sickness and headache, after adjusting for gender, age, self reported anxiety level, attention to the movie and show time.

**Conclusions:**

Seeing 3D movies can increase rating of symptoms of nausea, oculomotor and disorientation, especially in women with susceptible visual-vestibular system. Confirmatory studies which include examination of clinical signs on viewers are needed to pursue a conclusive evidence on the 3D vision effects on spectators.

## Introduction

Following the market expansion of movies filmed with three dimensional (e.g. 3D) technology and televisions equipped with 3D displays for the home entertainment, there has been an increasing concern about possible side effects on spectators. It has been suggested that the viewing of 3D stereoscopic stimuli can cause vision disorders to manifest in previously asymptomatic individuals [1], [2]. The American Optometric Association estimated that 3–9 million of Americans have problems in binocular vision and, consequently, in viewing 3D movies [2]. People with normal binocular vision should not be affected. In Europe, an advisory board set up by the Italian Ministry of Health concluded that “…the national or international literature shows no evidences that the vision of three dimensional movies forces eyes or brain to elaborate (visual) information in a non natural way” (Italian Ministry of Health, Circolare 12486/2010). Nevertheless, the prevalence of health outcomes on 3D movie spectators appears to be increasing in domestic environments [2], [3].

Previous research on professional exposures to virtual reality systems [4], vehicle simulators [5] and stereoscopic displays [6], [7] have reported that several adverse health effects can be induced by viewing motion images, including visual fatigue [7] (also termed asthenopia [8] or eyestrain [9]) and visually induced motion sickness (VIMS [5]).

Symptoms of visual fatigue induced by images comprise eye discomfort and tiredness, pain and sore around the eyes, dry or watery eyes, headaches and visual distortions such as blurred and double visions, and difficult in focusing (see [6] for a full review). The main physiological mechanism involved with the onset of visual fatigue concerns the intense eye accommodation activity of 3D movie viewers, such as focusing and converging. It has been argued [10] that eye focus cues (accommodation and blur in the retinal image) target the depth of the display (or of the movie screen) instead of the displayed scene, generating unnatural depth perception. Additionally, uncoupling between vergence and accommodation affects the binocular fusion of the image. Both processes may generate visual fatigue in susceptible individuals. Since data from symptomatic optometric clinic patients indicate prevalence between 9.2% (accommodative insufficiency) to 7% (convergence insufficiency; but see [11] for details), prevalence of visual fatigue induced by 3D movie in susceptible spectators might also be large.

In addition to symptoms of visual fatigue, viewers of 3D may experience nausea (nausea, increased salivation, sweating) and disorientation (dizziness, vertigo, fullness of head). Those symptoms are indicative of VIMS, a condition that may onset during or after viewing dynamic images while being physically still [5], when images induces in the stationary spectator a sense of vection (i.e. illusion of self movement). The most accepted explanation for VIMS is the classical conflict theory based on the mismatch between the visual, the proprioceptive and the vestibular stimuli [3], [12]. In this case, the visual system feels vection while the vestibular and proprioceptive systems do not transmit signals consistent with motion. Notably, although VIMS and visual fatigue are different conditions, they probably share some common biological mechanisms ([13], [14]).

More recently the specific disturbance deriving from viewing 3D movies has been named “3D vision syndrome” [2] but the relative occurrence of different symptoms in spectators and the individual characteristics that make some individuals more susceptible than others still remain to be described. Previous research showed that occurrence of self reported symptoms in young healthy adults during or immediately after watching a 3D movie may be high ([10], [15], [16]), although often quickly disappearing once they finished viewing. In this paper, I aim to compare the frequency and intensity of VIMS after viewing a traditional 2D movie with the ones after viewing a 3D movie and to assess the characteristics of susceptible individuals.

## Methods

### Study design and eligibility

A prospective carryover observational study was designed to assess the effect of 2D and 3D movie views on self reported symptoms by means of questionnaires. A convenience sample of healthy adult volunteers was enrolled during December 2011. For each individual, at day of enrollment, socio demographic and health related information was compiled and 2 before - after paper based questionnaires (see below) were provided. Within the following 3 weeks participants were asked to go in different days to a cinema to see a 2D and a 3D movie of their choice. Participant were also left free to chose what movie to see first (if 2D or 3D). Participants who watched no films, or only a single movie, or 2 movies of the same type (both 2D or both 3D) were excluded from the analysis.

### Ethic statement

The anonymous data collection procedures, in which subjects provided written informed consent, were approved by the Ethic Committee of the Sapienza University Hospital Policlinico Umberto I, Rome (Prot. 937/11).

### Questionnaire

The questionnaire was divided into three section. Section 1 included socio-demographic variables (gender, age, marital status, educational level, employment status), use of prescription glasses or contact lenses when watching movies and possible individual predictors of VIMS symptoms as history for headache, car sickness, vertigo disturbances (including other non specific forms of dizziness) and daily commitments to the use of computer and/or video games. Headache, car sickness and dizziness are all possible correlates with individual sensitivity of the visual-vestibular system to external stimuli. Long sessions of computer and/or videogames may increase the risk of asthenopia [17], as visual fatigue can accumulate with prolonged visual stress [6].

History for headache (“How often did you suffer because of headache in the last year”), car sickness (“How often did you suffer from car sickness when traveling by car on long journeys or bendy roads”), vertigo/dizziness (“How often did you suffer because of dizziness or vertigo in the last year”) were assessed with a 5 point Likert scale (never, almost never, sometimes, often, very often). The amount of time per day spent in front of a computer or a game console for work or leisure was assessed with a 3 point Likert scale (none, <5 hours, ≥5 hours).

Additionally, self perceived anxiety level (“Would you define yourself as an anxious person?”) was assessed with a 5 point Likert scale (never, almost never, sometimes, often, very often). Self perceived anxiety level was taken into account for interaction between psychological and physiological processes within everyday situations. For example, anxiety level might be connected with increased symptom reporting in some individuals.

The standardized Simulator Sickness Questionnaire (SSQ; [18]) made up the two other sections of the questionnaire. The SSQ is composed of a 16-item symptom checklist and each item is rated from none to severe (scored respectively from 0 to 3). Total SSQ score is obtained by adding symptom scores multiplied by 3.74. Total severity scores greater than 20 indicate participants are experiencing sufficient discomfort, scores less than 5 indicate symptoms are negligible.

Notably, SSQ can be split into 3 subscales: nausea, oculomotor and disorientation. Scores on the nausea subscale, represent symptoms related to gastrointestinal distress which are associated with the autonomic nervous system (e.g., nausea, stomach awareness, and burping [18]. Scores on the oculomotor subscale, reflect symptoms related to disturbances of the visual system and included symptoms associated with vision (e.g., difficulty focusing, blurred vision) and visual fatigue (e.g., eyestrain, headache). Scores on the disorientation subscale are related to disturbances of the vestibular system (e.g., dizziness, vertigo).

The SSQ symptom checklist was compiled before and after each movie. Therefore, 4 SSQ symptom lists (before and after the 2D movie, before and after the 3D movie) were collected for each participants. The before movie scores provided the baseline conditions of participants before each movie.

Information on the movie title, time of vision (afternoon, first evening or late evening show time), the closeness of the seat to the movie screen (if within the first 3 rows or otherwise) and the attention devoted to the film (Likert 5 points scale from low attention to very high) were also collected.

### Statistical analysis

Pre and post exposure symptom frequencies were compared by estimating crude Odd Ratio (OR) with relative 95%CI separately for 2D and 3D movies. Since SSQ provides reliable assessment only for healthy individuals at baseline, those individuals reporting total scores>15 before the 2D or the 3D movie viewing were not considered in this analysis (respectively 64 and 46 individuals).

Formal analysis of the cross over design was done using a mixed model. In this model the response variable SSQ total score was dichotomized using a cut off of 15. Exposure (3D vs 2D movie vision), individual and movie vision characteristics were modeled as fixed effect while individuals were modeled as random effect. For this analysis, the individual and movie vision characteristics were dichotomized as described below. Responses were coded as 1 for: often and very often headache, car sickness, vertigo/dizziness and anxiety; glasses or contact lenses use at cinema; >5 hours per day use of computer or game console; ≥22.30 movie starting time; no and little movie enjoyment; sit in front rows. All other responses were coded as 0. Before the analysis, a correlation analysis was ran among predictor variables to avoid variance inflation of the subsequent model. The analysis was carried out with the software R (freely available at http://cran.r-project.org) using the function *glmer* available with the package *lm4* [19], [20].

## Results

### Characteristics of the study population

In total 524 participants were enrolled in the study. Of those, 20 individuals saw a single movie, 2 individuals saw two 2D movies but no 3D movie, 5 individuals saw two 3D movies but no 2D movie and were excluded from the analysis. The final sample was composed by 497 individuals (Table 1).

**Table 1:** Socio-demographic and individual characteristics of study participants (N = 497).

Variable		(%)
Age	<20	7.6
	20–29	69.0
	30–39	14.9
	40–49	2.4
	> = 50	6.0
Gender	Female	55.7
	Male	44.3
Marital status	Married (or co-habitant with partner)	19.5
	Divorced or widowed	1.6
	Never married (nor currently cohabitant)	78.9
Educational level	<High school	6.4
	High school	48.3
	University level degree	45.2
Employment	Currently employed full time	31.6
	Currently employed part time	7.0
	Currently unemployed or retired	11.1
	University student	50.3
Use of glasses or prescription lenses at cinema	No	55.5
	Yes, glasses	29.2
	Yes, lens	15.3
Headache in a typical month	Very often	6.6
	Often	12.3
	Sometimes	30.8
	Almost never	35.6
	Never	14.7
Car sickness when traveling by car on long journeys or bendy roads	Very often	6.0
	Often	8.9
	Sometimes	23.9
	Almost never	27.0
	Never	34.2
Dizziness or vertigo in a typical month	Very often	0.6
	Often	5.0
	Sometimes	20.9
	Almost never	30.6
	Never	42.9
Self perceived high anxiety level	Very often	5.4
	Often	13.5
	Sometimes	29.2
	Almost never	27.6
	Never	24.3
Daily use of computer and/or video game console	<1 hour	5.8
	1–5 hors	58.6
	>5 hours	35.6

Participants were between 18 and 65 years of age, with more than half of them (54.1%) between 18 and 25 years of age. Females (55.9%) were slightly more than males. Most responders were never married nor currently cohabitant with a partner (78.9%) and university students (49.9%).

Less than half of participants used visual corrections: 29.2% wore glasses and 15.3% contact lenses when watching a movie at cinema. In a typical month, 18.9% of participants have headaches often or very often, 14.9% often or very often have of motion sickness when traveling by car on long journeys or bendy roads, 6.0% often or very often have dizziness/vertigo, 18.1% define him or herself as an anxious person, 35.6% report to use of computer and/or video game console for more than 5 hours per day.

### Characteristics of the vision

Among the 2D movies seen, the most frequently reported were New years Eve (11.3%), Finalmente la felicità (15.7%), Immaturi – il viaggio (12.3%), Midnight in Paris (7.0%), Sherlock Holmes: a game of shadows (19.9%), Vacanze di Natale a Cortina (14.9%). The preferred time of viewing the 2D movies was the late evening (44.1%), followed by the early evening (36.4%) and the afternoon (19.5%) show times and 6% of individuals were sitting close to the screen (Table 2). Attention to the 2D movie was high or very high in 87.3% of individuals.

**Table 2:** Movie vision characteristics and occurrence (%) of Simulator Sickness Questionnaire (SSQ, [18]) subscale scores after the 2D and the 3D movies.

Variable		2D (N = 433)	3D (N = 451)
Position during vision: proximity to the screen	Sit close to movie screen	6.0	4.8
	Other	94.0	95.2
Showtime (movie starting time)	Before 20.30	19.5	29.6
	20.30–22.29	36.4	39.6
	22.30 or later	44.1	30.8
Attention/involvement to the movie	Very low	0.0	0.2
	Low	2.4	3.8
	Moderate	10.3	15.9
	High	46.7	47.3
	Very high	40.6	32.8
Nausea score	0	78.5	53.9
	1	14.8	22.6
	2	4.6	12.9
	3	1.2	5.5
	> = 4	0.9	5.1
Oculomotor score	0	52.9	19.3
	1	23.8	14.2
	2	11.5	21.5
	3	6.9	10.9
	> = 4	4.8	34.1
Disorientation score	0	75.3	37.7
	1	19.4	24.2
	2	3.0	16.4
	3	1.8	8.0
	> = 4	0.5	13.7
Total SSQ score	0–14	85.9	45.2
	> = 15	14.1	54.8

Individuals having SSQ total score>15 before the movie viewing were excluded from the analysis.

Regarding the 3D movies, most participants saw Puss in boots (79.9%) or Arthur Christmas (8.7%). The 3D movie goers were distributed between the afternoon (29.6%), the first evening (39.6%) and the late evening show times (30.8%) and 4.8% of individuals were sitting close to the screen (table 2). Attention to the 3D movie was high or very high in 80.1% of individuals.

### Frequency of symptoms and association with individual and movie vision characteristics

**Figure 1:**
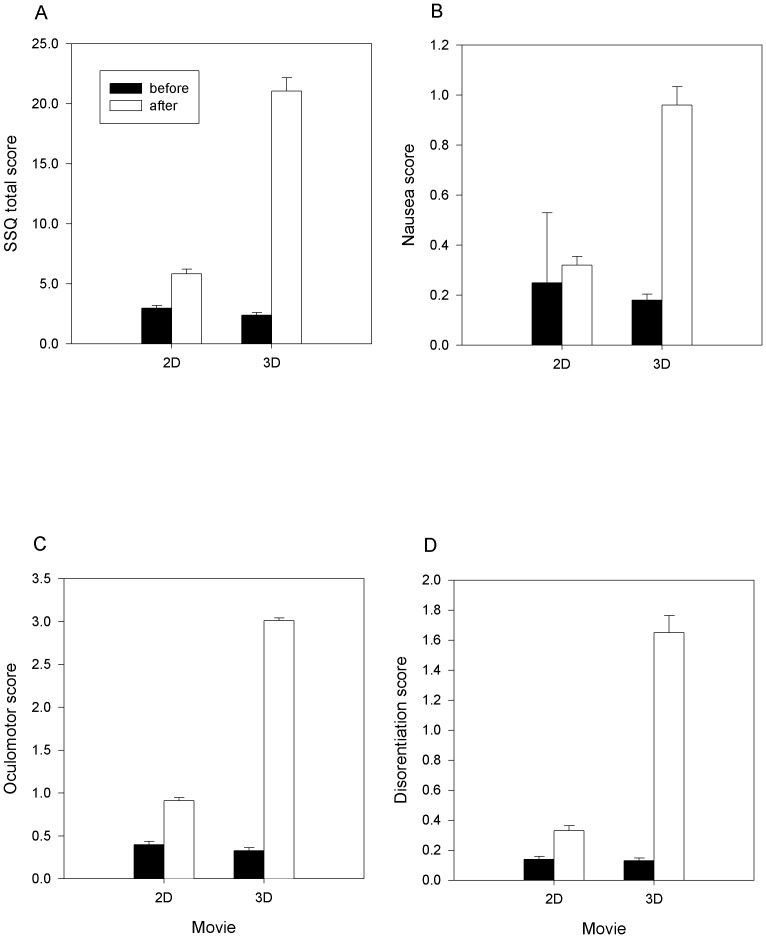
Intensity of visually induced motion sickness before and after viewing 2D and 3D movies. Mean (95% CI) scores of total SSQ (A), Nausea (B), Oculomotor (C) and Disorientation (D) subscales are shown (SSQ: Simulator Sickness Questionnaire [18]).

Mean SSQ total score and the mean scores of the 3 subscales (nausea, oculomotor and disorientation) increased significantly after the 2D and the 3D movies (Figure 1A–1D; paired t-test, all comparisons p<0.05). Total SSQ score increased almost 2 times from baseline after watching a 2D movie but increased 8.8 times after watching a 3D movie (Figure 1A). Similarly after the 3D movie, the increases of nausea, oculomotor and disorientation mean scores were respectively 5.3, 9.1 and 12.7 times above baselines, compared to the increases of respetively1.3, 2.2 and 2.3 times above baselines reported for the 2D movie (Figure 1B–1D). Post exposure total score SSQ>15 increased from 14.1% after the 2D movie to 54.8% after the 3D movie (table 2).

Movie watchers reporting a post exposure total score SSQ>15 were 14.1% after the 2D movie, while were 54.8% after the 3D movie (Table 2). Differences between 2D and 3D movies of post exposure scores were also evident for the SSQ subscales. After the 3D view, 10.6% of participants had a score of 3 or more in the nausea subscale (compared to 1.1% after the 2D view), 45.0% of participants had a score of 3 or more in the oculomotor subscale (compared to 11.7% after the 2D view), 21.7% of participants had a score of 3 or more in the disorientation subscale (compared to 2.3% after the 2D view).

**Figure 2:**
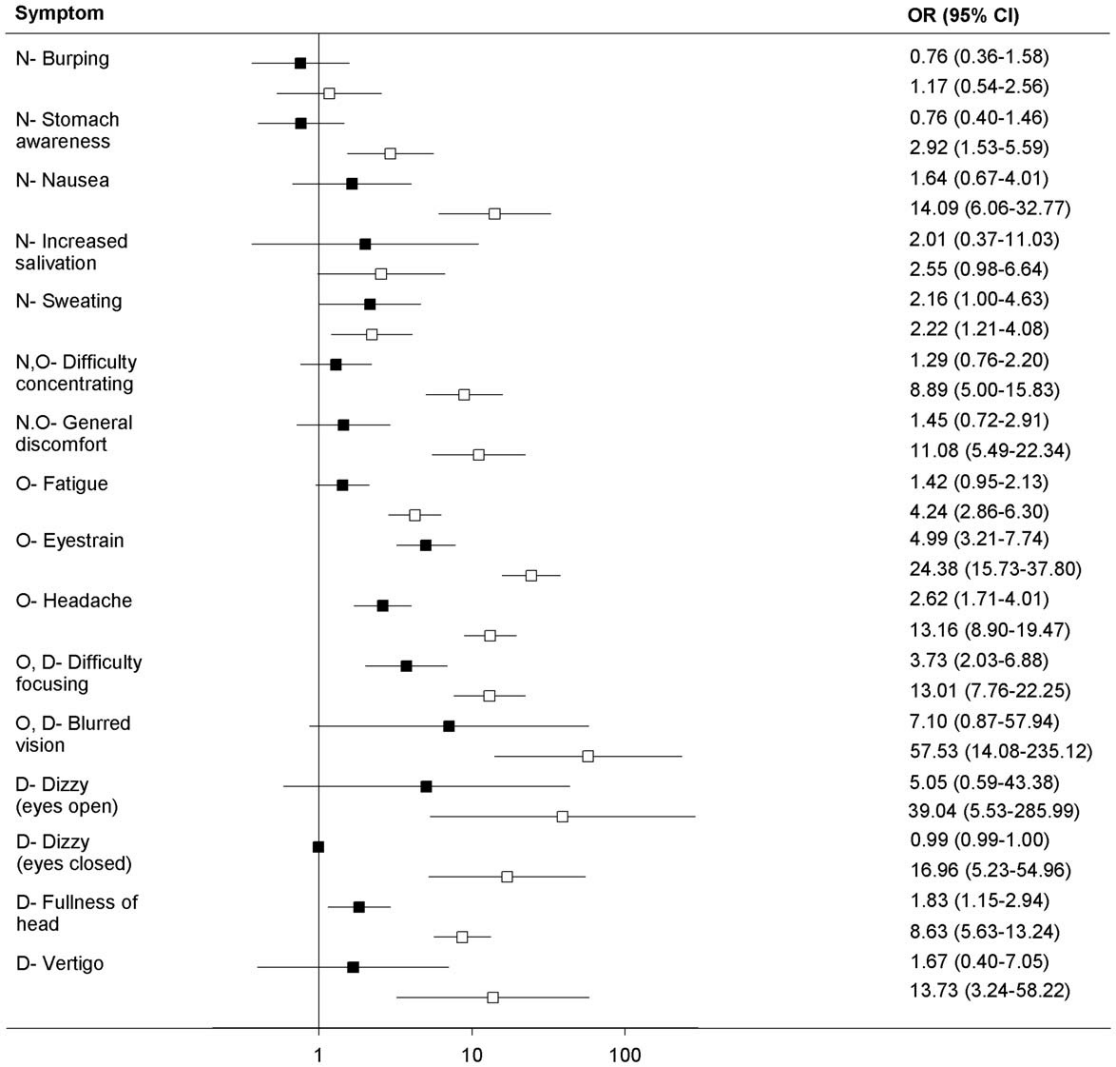
Crude odds ratios (95% CI) of symptom frequency of movie goers. Closed quadrates: 2D movies; open quadrates: 3D movies. Symptoms belong to Nausea (N-), Oculomotor (O-) and Disorientation (D-) subscales of the Simulator Sickness Questionnaire [18]. Some symptoms belong to two subscales.

The crude odds ratios (Figure 2) of onset of single symptoms resulting from exposure to the 2D movie ranged between 0.76 (burping) and 4.99 (eye strain). Significant effects were detected for 3 symptoms belonging to the oculomotor section of SSQ: eyestrain (OR = 4.99; 95%IC = 2.31–7.74), difficulty focusing (OR = 3.73; 95%IC = 2.03–6.88) and headache (OR = 2.62; 95%IC = 1.71–4.01) and for fullness of head (OR = 1.83; 95%IC = 1.15–2.94), but not in all the others.

The crude odds ratios (Figure 2) of onset of single symptoms resulting from exposure to the 3D movie ranged between 1.17 (burping) and 57.53 (blurred vision). Significant effects were detected for all symptoms in the SSQ list with the exception of burping (OR = 1.17; 95%IC = 0.54–2.56) and increased salivation (OR = 2.55; 95%IC = 0.98–6.64). Higher values in the SSQ nausea section resulted for nausea (OR = 14.09; 95%IC = 6.06–32.77), in the oculomotor section for eyestrain (OR = 24.38; 95%IC = 15.73–37.80) and in the disorientation section for Dizzy (eyes open) (OR = 39.04; 95%IC = 5.33–285.99).

Multivariate analysis on total SSQ score (as binomial term with cutoff at score = 15) was ran using a mixed model (Table 3). Treatment effect (3D movie) was highly significant (b = 2.84±0.27, p<0.001) as well as the effects of car sickness (b = 0.95±0.33, p<0.01) and headache (b = 1.01±0.31, p<0.01). Among the predictors, female gender was positively correlated with car sickness, headache and vertigo (Pearson correlation, all p<0.001).

**Table 3:** General linear model of total Simulator Sickness Questionnaire score (as binomial term with cutoff at SSQ = 15).

Variable	Coefficient	Standard error	P value
Exposure (3D movie vs 2D)	2.85	0.27	<0.001
Time order	0.04	0.22	0.847
Carsickness often and very often	0.95	0.33	0.004
Headache often and very often	0.99	0.31	0.001
Use of computer or videogame console>5 h per day	0.22	0.25	0.383
Use of prescription glasses	0.30	0.27	0.260
Use of prescription lenses	0.24	0.34	0.490
Sit close to screen	0.79	0.25	0.383
Use of computer or videogame console>5 h per day	0.22	0.25	0.383

The model is adjusted for gender, age, self reported anxiety level, attention to the movie and show time. Individuals having SSQ total score>15 before the 2D or the 3D movie visions were excluded from the analysis.

## Discussion

Our analysis suggests that viewing a 3D movie commonly produces visual symptoms. It should be noted that these are self reported symptoms of transient mild discomfort that usually disappear when 3D glasses are removed. If we use the cutoff of SSQ total score at 15, more than half of participants (52.5%) reported the outcome. Using a more conservative cutoff (SSQ total score>20), 38.1% of participants reported the outcome. Pre -post exposure changes of SSQ total score is larger after a 3D movie (8.8 times higher than baseline) than after a 2D movie (2 times higher the baseline). A similar result was observed by [15] in the viewers of a single 3D movie (“U2 3-D”), although symptom severity was relatively lower than in our study and often related to the oculomotor scale only. In our study, crude odd ratios higher than 5 resulted for almost all oculomotor and disorientation related symptoms, with eyestrain peaking at a remarkable value of OR = 15.24 (95%IC: 10.76–21.60). Such high rate of symptom onset could be related to the larger sample used in our analysis that increased the likelihood that susceptible individuals could by chance be present in the study population. Given the current prevalence of vision disorders in the general population ([11]), it is possible that 3D viewing cause those problems to manifest in asymptomatic individuals ([1]).

Although the assessment of the specific mechanisms that caused the increase of VIMS in spectators of the 3D movies were beyond the scope of our study, the adopted research design allowed participants to be exposed to a variety of commercially available 3D films. Factors that are reported to be associated with VIMS can be categorized into (i) factors associated with the visual stimuli provided to viewers, (ii) factors associated with the position from where the viewers are watching the movie and (iii) the psychophysiological conditions of the viewers. Examples reported in literature include (but are not limited to): the characteristics of the (moving) images (e.g. the optic flow) such as the earth axis along which the visual field is made rotating ([21], [22]), the amplitude of the field of view ([23]), the display angle ([24]), the feeling of immersion or presence ([25]), the (co-)presence of vection ([26], [27]), the display types ([7]), postural instability ([28], [29]), habituation ([30]), age ([30]), gender ([31]), and anxiety levels of viewers ([32]), and others. Interactions and additive effects among factors may also be present, making difficult to predict the final outcome (if a given individual will or will not suffer VIMS).

Earlier experiences of visual discomfort observed in 3D display viewers [9], [33],[34],[35] led to the hypothesis that the conflict between vergence and accommodation stimuli is the cause of such visual discomfort ([33], [35], [36]). Controlled experimental conditions in which the effect of the vergence-focal conflict on visual fatigue could be isolated from other variables confirmed such explanation [10]. More recently [37] sorted out experimentally the factors involved in the vergence-accomodation conflict by manipulating the viewing distance, vergence distance, the type of disparity (crossed or uncrossed) and the focal distances and provided some guidelines to minimize viewers discomfort. Additionally, it has been argued that 2D movie viewers tend to focus at the actors while the eye movement patterns of 3D viewers are more widely distributed to other targets such as complex stereoscopic structures and objects nearer than the actors [38]. This behavior might increase the vergence-accomodation mismatch, increasing the visual stress on 3D spectators. The higher intensity of visual symptoms when participants were exposed to the 3D movie compared to the 2D movie observed in our study could be taken as a large scale evidence of such hypothesis.

Possibly, a partially different mechanism is involved in the onset of nausea and disorientation related symptoms [14]. Nausea, dizziness and vertigo are connected to vestibular disturbance and the visual – vestibular interactions and the classical sensory conflict theory ([39]) can explain the onset of symptoms in susceptible individuals.. The public health relevance of VIMS was raised some years ago in Japan when 36 (out of 294) high school students were hospitalized for motion sickness after watching a movie characterized by unexpected whole image motion and vibration (the so called Matsue movie sickness incident [40]). In our study, disorientation symptoms are not reported after the 2D movies and they seem typical side effects in spectators of the 3D movies. Those symptoms have not been included so far in the protocols of studies on exposure to 3D visual stimuli [8], but we claim for their inclusion in future studies as they represent a large and peculiar portion of total 3D induced discomfort.

Multivariate analysis suggests that seeing a 3D movie increases SSQ scores. Besides the exposure to 3D, significant predictors of higher SSQ total score were car sickness and headache after adjusting for gender, age, self reported anxiety level, attention to the movie and show time. The use of glasses or contact lenses does not seem to increase the risk of raising SSQ scores. Women with a history of frequent headache, carsickness (and possibly dizziness, which is correlated with the above mentioned variables) may be more susceptible to VIMS than others. The relationships between motion sickness, vertigo, dizziness and migraine is well documented ([41],[42],[30]), and 3D movies may interact with these conditions to produce more symptoms than 2D movies.

The crossover design of this research accounted for confounding differences in the composition of the two groups being compared as would have been resulted from cohort type designs. It should be noted that, given the non experimental nature of the study design, the 2D and 3D movies viewed by a given individual were not the same. Therefore, differences in symptom frequency might be confounded by factors other then stereoscopic view and not included in the covariates assessed here. Potential limitation of our study include the small range in age of participants (most of them were between 18 and 30) which limits the ability to extend conclusions to older age groups. Assessment of the visual effects of 3D movies in children and older adults appear warranted. Another possible methodological limitation regards the impossibility of masking the exposure. Individuals knew if they were watching a 2D or a 3D movie and some of them might have reported higher rates of symptoms because of autosuggestion. Although we corrected for the self reported anxiety level of individuals, as more anxious persons are probably those reporting more symptoms than actually suffered, only future experimental studies based on clinically assessed signs could fully avoid this potential bias.

## Conclusion

Viewing 3D movies can increase rating of nausea, oculomotor and disorientation. Analogous to riding a roller coaster, for most individuals the increases in symptoms is part of the 3D experience and enjoyment and these experiences is not necessarily an adverse health consequence. However, some viewers will have responses that in other contexts might be unpleasant. In particular, women with susceptible visual-vestibular system may have more symptoms when watching 3D movies. Individual variability of the 3D exposure including the length of the movie, the angle of view and the pre exposure baseline conditions are potential predictors of visual discomfort that may warrant future investigation. As noted by others, 3D viewing may increase task burdens for the visual system, and susceptible individuals may develop a “3D vision syndrome” [1]. Due to increasing commercial releases of 3D movies and displays for home and professional use it is likely that more people will complain about these symptoms. For those with symptoms a simple optometric visit might be advisable [1].
